# Calcineurin stimulation by Cnb1p overproduction mitigates protein aggregation and α-synuclein toxicity in a yeast model of synucleinopathy

**DOI:** 10.1186/s12964-023-01242-w

**Published:** 2023-08-24

**Authors:** Srishti Chawla, Doryaneh Ahmadpour, Kara L. Schneider, Navinder Kumar, Arthur Fischbach, Mikael Molin, Thomas Nystrom

**Affiliations:** 1 Institute for Biomedicine, Sahlgrenska Academy, Centre for Ageing and Health – AgeCap, University of Gothenburg, Gothenburg, 405 30 Sweden; 2 Department of Life Sciences, Chalmers University of Technology, Gothenburg, Sweden; 3 Center for Bionics and Pain Research, Sahlgrenska University Hospital, Mölndal, 431 30 Sweden; 4 Max Planck Institute for Biology of Ageing, Cologne, 50931 Germany

**Keywords:** α-synuclein, Calcineurin, Protein phosphatase 2B, Protein aggregation, Protein quality control

## Abstract

The calcium-responsive phosphatase, calcineurin, senses changes in Ca^2+^ concentrations in a calmodulin-dependent manner. Here we report that under non-stress conditions, inactivation of calcineurin signaling or deleting the calcineurin-dependent transcription factor *CRZ1* triggered the formation of chaperone Hsp100p (Hsp104p)-associated protein aggregates in *Saccharomyces cerevisiae*. Furthermore, calcineurin inactivation aggravated α-Synuclein-related cytotoxicity. Conversely, elevated production of the calcineurin activator, Cnb1p, suppressed protein aggregation and cytotoxicity associated with the familial Parkinson’s disease-related mutant α-Synuclein A53T in a partly *CRZ1*-dependent manner. Activation of calcineurin boosted normal localization of both wild type and mutant α-synuclein to the plasma membrane, an intervention previously shown to mitigate α-synuclein toxicity in Parkinson’s disease models. The findings demonstrate that calcineurin signaling, and Ca^2+^ influx to the vacuole, limit protein quality control in non-stressed cells and may have implications for elucidating to which extent aberrant calcineurin signaling contributes to the progression of Parkinson’s disease(s) and other synucleinopathies.

Video Abstract

**Supplementary Information:**

The online version contains supplementary material available at 10.1186/s12964-023-01242-w.

## Background

The calcineurin phosphatase (CN), also called PP2B, is an evolutionary conserved protein belonging to the PPP3 class of serine-threonine phosphatases [1], which requires Ca^2+^ for its activity. Even minor changes in the exquisitely low cytosolic Ca^2+^ levels detected in unstimulated cells trigger distinct spatial and temporal cellular responses orchestrated and integrated by CN [1–6]. Apart from Ca^2+^ homeostasis, diverse pathways, including cell cycle control, pH homeostasis, and stress-activated transcriptional regulation are controlled by CN through its specific interaction with different substrates [6], relying on different substrate-binding docking motifs [3, 6–8].

CN is a heteromer encoded by *CNA1* (or *CNA2*/*CMP*2) and the regulatory subunit encoded by *CNB1*. This complex acts downstream of the calmodulin effector molecule in response to Ca^2+^ binding. The association between calmodulin and CN releases the autoinhibitory domain from CN in the holoenzyme complex. The activated CN complex adjusts several fundamental cellular activities/features, including development, behavior, lifespan, and adaptive responses that are either dependent or independent of the downstream transcription factor *CRZ1* [9–16]. CN-dependent Ca^2+^ homeostasis also involves multiple organelles and its dysregulation is known to cause problems also in protein homeostasis (proteostasis), including α-synuclein (α-Syn) proteinopathy related to Parkinson’s disease [17–20]. In addition, Habernig et al. [21], recently demonstrated that the cytotoxicity of α-Syn could be suppressed by administering extracellular Ca^2+^ and that this limited the oligomerization and toxicity of α-Syn by boosting lysosomal protease cathepsin D activity in a CN-dependent manner.

So far, the relationship between CN/Ca^2+^ signaling and Protein Quality Control (PQC) is not well understood. To elucidate the possible participation of CN and CN-dependent regulators, such as Ca^2+^ transporters/channels and downstream substrates of CN in PQC, we investigated to which extent such factors affected the generation and management of cellular protein aggregates in the yeast *S. cerevisiae*. We show that reduced CN signaling, through the deletion of the regulatory subunit *CNB1*, or deleting both the catalytic subunits (*CNA1* and *CMP2*), during steady-state, non-stress conditions in Ca^2+^-replete medium triggers the formation of Hsp100p (Hsp104p)-associated protein aggregates and aggravates the cytotoxicity of both wild-type alpha-synuclein (α-Syn) and mutant α-Syn-A53T, associated with early-onset familial Parkinson’s disease. In contrast, enhanced Cnb1p production not only suppressed Hsp104p-associated protein aggregation but also supported growth in the presence of the hypertoxic α-Syn-A53T mutant allele. These effects were dependent, in part, on the transcription factor *CRZ1* acting downstream of CN. The data suggest an intimate relationship between CN signaling and proper PQC and that CN/Ca^2+^ signaling limits proteostasis during normal steady-state growth under non-stress conditions and counteracts proteotoxicity associated with synucleinopathies.

## Results

### Altering calcineurin signaling in non-stressed cells affects protein aggregation

**Fig. 1:**
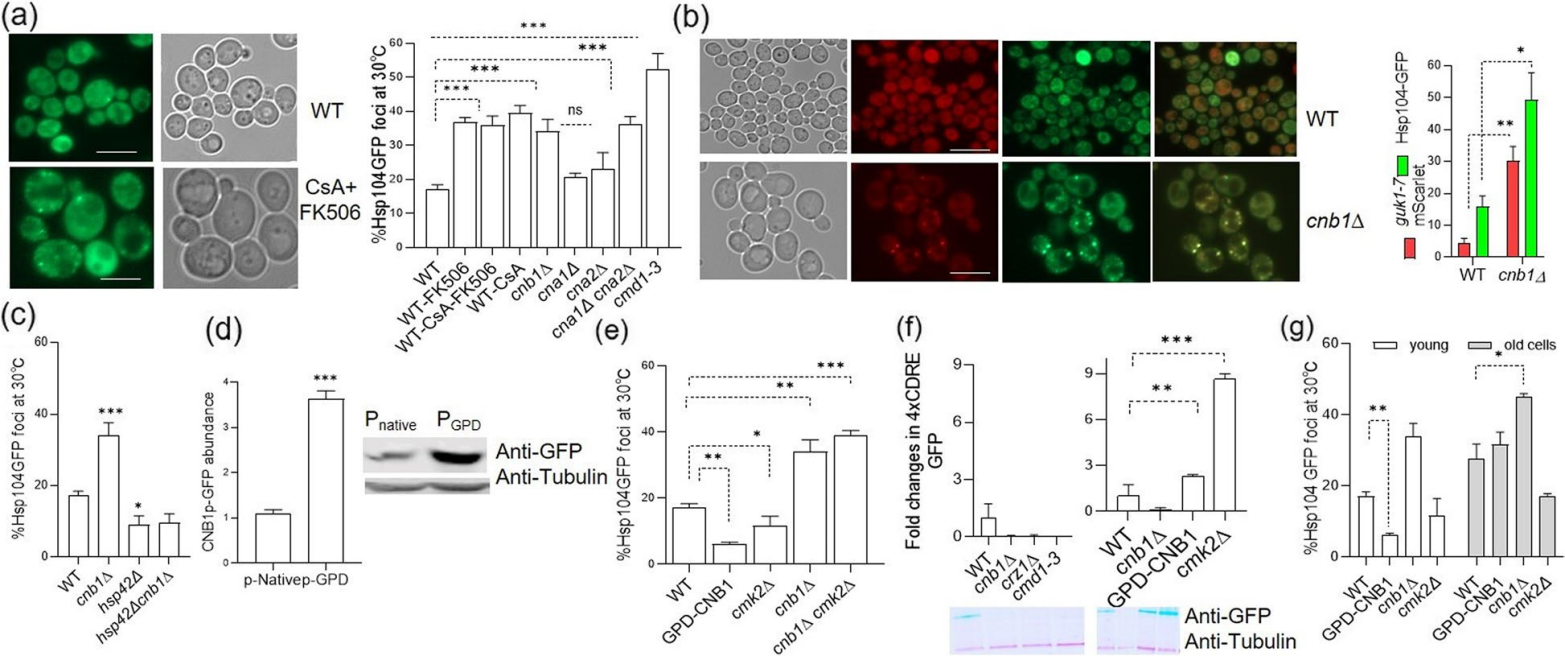
Effects of altered calcineurin activity on protein quality control. **a** Yeast cells expressing Hsp104p-GFP from its endogenous locus, treated as indicated with 1 µg/ml concentration of calcineurin inhibiting drugs FK506 and CsA for 30 min. Representative fluorescence microscopy images are displayed. The percentage of cells showing Hsp104p-GFP associated aggregates in WT, *cna1*∆, *cna2*∆, *cnb1*∆, and the double mutant *cna1*∆ *cna2*∆ are shown. **b** Representative images WT and *cnb1*∆ cells expressing Hsp104p-GFP (green) and guk1-7p-mScarlett (red). The *cnb1*∆ mutant accumulated more of Hsp104p-GFP (green) and guk1-7p-mScarlett associated aggregates of the reporter proteins than WT as quantified along with the microscopic images. **c** Quantification of the percent cells containing Hsp104p-GFP associated aggregates in WT cells and cells devoid of *cnb1∆, hsp42*∆, and *cnb1*∆ and *hsp42*∆ are shown. **d** Immunoblot analysis of Cnb1p-GFP levels regulated by the native or GPD promoter. Blot was probed with anti-GFP antibody and as a loading control a yeast anti-tubulin antibody. **e** Suppression of Hsp104p-GFP associated aggregates formation upon CNB1 overexpression. Total number of cells with aggregates shown for WT, GPD-CNB1, *cmk2*∆ and *cnb1∆ cmk2*∆ cells. **f** Immunoblot analysis of protein extracts from WT cells and the indicated deletion mutants to measure relative CN activation via the reporter plasmid pAMS366, expressing GFP under the control of 4X-CDRE [29]. Quantification of GFP vs. yeast tubulin protein levels as indicated (*n* = 3). **g** Quantification of Hsp104p-GFP associated aggregates indicated for various mutants in the young and aged population of WT, *GPD-CNB1*, *cmk2*∆ and *cnb1∆ cmk2*∆ cells. Quantitative data shown are averages of 3 independent samples and error bars indicate SD, where 200 cells were counted for each independent experiment. The scale bar represents 5 μm. *p < 0.05, **p < 0.005,***p < 0.0005, unpaired two-tailed t test

To study the consequences of calcineurin (CN) inhibition on protein quality control in yeast cells growing under non-stress conditions in Ca^2+^-replete media, we assessed the aggregation of cytosolic proteins using the yeast disaggregase Hsp104p fused to GFP as a reporter [22, 23]. In addition, we used the misfolding-prone allele of the yeast guanylate kinase enzyme, guk1-7p, tagged with mScarlett [24, 25] as a more direct reporter for misfolding [26]. Growing WT cells were treated with a 1 µg/ml concentration of the immunosuppressant drugs FK506 and CsA (Cyclosporin A) for 30 mins to inactivate CN. Cells treated with these drugs displayed increased protein aggregation as numerous Hsp104p-GFP foci formed in the cytosol in an enlarged fraction of cells (Fig. 1a). Next, we tested the effect of genetic deletions of individual subunits of the dimeric CN holoenzyme to assess the contribution of each subunit to proteostasis. For this, we created mutants defective in the catalytic subunits (*cna1∆*, *cna2*∆/*cmp2*∆) and the regulatory subunit (*cnb1∆*) carrying genomic Hsp104p-GFP. We found that deleting the redundant catalytic phosphatase subunits individually had little effect on aggregation while deleting both substantially increased the level of Hsp104p-GFP-associated aggregates (Fig. 1a). To further test whether reduced CN activity is encumbering PQC, we tested the Calmodulin allele *cmd1-3*, which encodes a mutant form of calmodulin that fails to bind Ca^2+^ and, therefore, cannot stimulate calcineurin activity [3, 6, 27, 28]. As expected, the strain showed much higher levels of cytosolic Hsp104p-GFP-associated protein aggregates than the WT strain (Fig. 1a). In addition, cells devoid of the regulatory subunit *CNB1* displayed a more than three-fold increase in the fraction of cells containing Hsp104p-associated aggregates (Fig. 1a) as well as a six-fold increase in guk1-7p aggregates (Fig. 1b). Additionally, the guk1-7p and the Hsp104p associated aggregates were found to co-localize in *cnb1*∆ (Fig. 1b).

It has been shown that the deposition of aberrant proteins into peripheral aggregates, namely IPODs and CytoQs, require the aggregase Hsp42p for their formation [24, 30–32] and we found that Hsp104p-associated aggregates formed upon defects in CN signaling (*cnb1*∆) required the presence of *HSP42* (Fig. 1c). The data suggest that a constitutive level of CN signaling is required in cells neither starving for Ca^2+^ nor being subjected to Ca^2+^ overload, to avoid the formation of Hsp42p-dependent aggregates (IPODs and CytoQs) formation.

We next asked whether CN signaling is limiting for PQC in otherwise wild-type cells by expressing *CNB1* under the control of the strong GPD promoter. Replacing the promoter resulted in a 3.5-fold increase in Cnb1p-GFP protein levels as detected by immunoblotting (Fig. 1d) and this overproduction markedly reduced Hsp104p-associated protein aggregation in wild-type cells (Fig. 1e). Another way to increase CN signaling is to delete *CMK2*, encoding a Calmodulin-dependent protein kinase acting as a negative regulator of CN signaling [33]. Deleting *CMK2* resulted in a reduction in protein aggregation, albeit more modest than by Cnb1p overproduction (Fig. 1e). The reduction in aggregation achieved by deleting *CMK2* required the presence of *CNB1* (Fig. 1e) demonstrating that the effects of deleting this negative regulator act through CN signaling. To measure cellular calcineurin activity, we used a reporter plasmid containing 4X-CDRE-GFP (Calcineurin Dependent Response Element) [29] and observed a nearly 8-fold increase in CDRE-GFP levels in *cmk2∆* and a more than 2.2-fold increased by overexpressing *CNB1* (Fig. 1f). As expected, the mutants *cnb1∆* and *cmd1-3* caused a drastic decrease in CDRE-GFP levels (Fig. 1f) confirming that the deletions and genetic modifications made generated the expected outcome in terms of CN signaling. Our observations collectively indicate that constitutive CN signaling under normal, non-stress, growth conditions is required to avoid the formation of Hsp42p-dependent aggregates and that boosting such CN signaling by overproducing Cnb1p can further improve PQC.

To investigate a possible role of CN activity also on aggregate formation during ageing we isolated young and old cells of wild-type and CN-affected mutant strains, as previously described [34, 35]. As seen in Fig. 1g, aged cells lacking *cnb1∆* accumulated more aggregates than the WT control but the difference between the strains was more evident in young cells. Similarly, constitutive overexpression of *CNB1* suppressed the Hsp104p-GFP-associated aggregate formation in young cells but failed to do so in aged cells (Fig. 1g). In contrast, cells lacking *cmk2∆* also suppressed age-associated aggregate formation (Fig. 1g). The data suggest that CN activation by Cnb1p overproduction and *cmk2∆* leads to quantitatively different effects on CN activity that may cause qualitatively different outcomes on CN output functions and/or effects on somewhat different pathways (see Fig. 1f and below).

### Cellular Ca^2+^ channels influence proteostasis differently

**Fig. 2:**
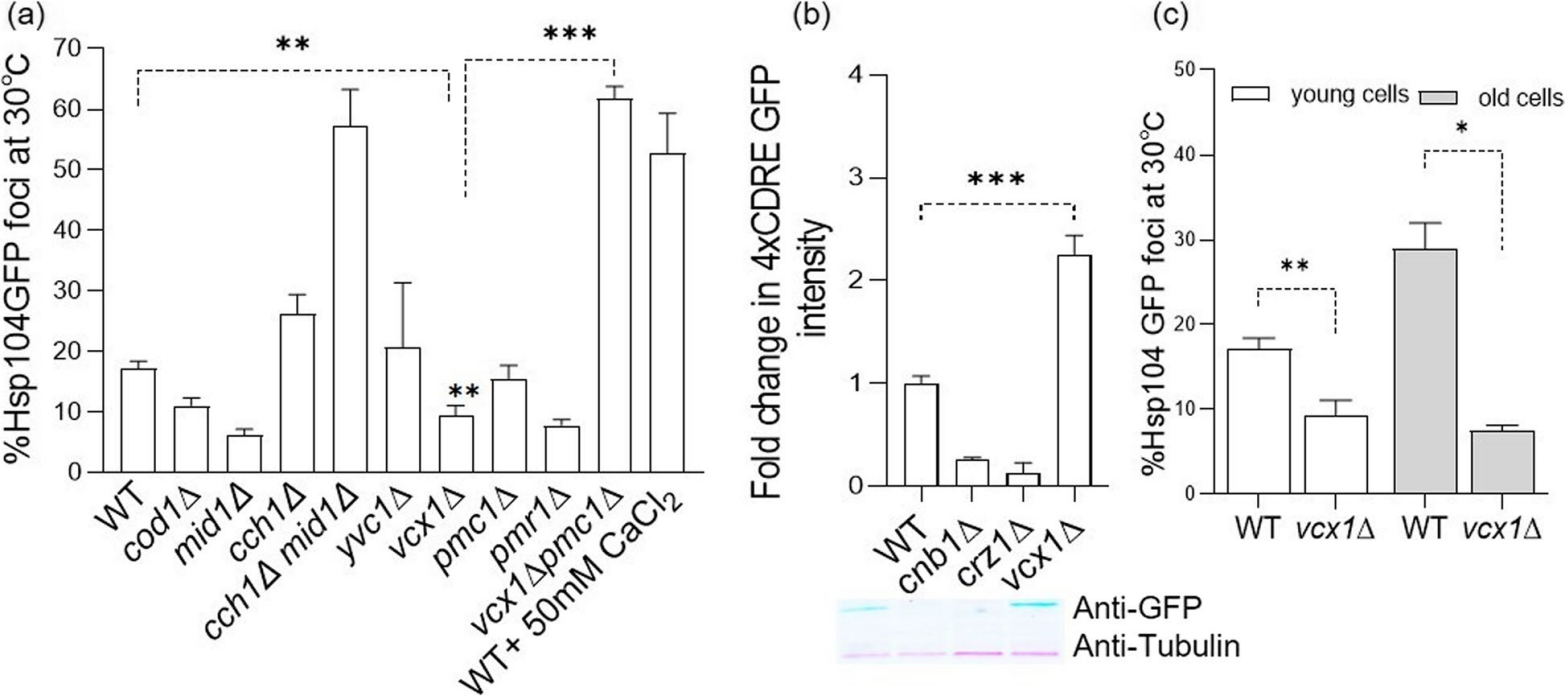
Influence of different Ca^2+^ channels on proteostasis. **a** Quantification of the percent cells containing Hsp104p-GFP associated aggregates in WT cells and various strains deficient in calcium regulatory channels as indicated. Results in WT cells treated with 50 mM CaCl_2_ for 30 min are also shown. **b** Immunoblot analysis of protein extracts from WT, *cnb1Δ, crzΔ*, and *vcx1Δ* cells expressing pAMS366 4X-CDRE-GFP [29]. Quantification of GFP vs. yeast tubulin protein levels is indicated (*n* = 3, ± SD). **c** Quantification of the percent cells containing Hsp104p-GFP associated aggregates in the young and aged population of WT and vcx1Δ cells. Data shown are the average of 3 independent ± SD. 200 cells from each independent experiments were counted and error bars indicate SD. *p < 0.05, **p < 0.005,***p < 0.0005, unpaired two-tailed t test

Basal [Ca^2+^] and CN signaling in the cell are maintained with the help of different Ca^2+^ regulating channels and pumps [36, 37]. Having established that some basal CN activity is critical for proper PQC we next determined which Ca^2+^ channel(s)/pump(s) are required in yeast cells to avoid protein aggregation under non-stress conditions. Specifically, Mid1p and Cch1p are two channels that work in parallel to provide Ca^2+^ from the cell exterior and we found that deleting both, as expected, resulted in an increased aggregation of cytosolic proteins, whereas individual deletions in the corresponding genes did not (Fig. 2a). It is unclear why *mid1∆* resulted in a reduced aggregation but this could be due to a compensatory up-regulation of calcineurin activity and/or other not yet identified Ca^2+^ influx pathways contributing to Ca^2+^ homeostasis. The ER/Golgi-resident Ca^2+^/Mn^2+^ ATPase, Pmr1p, and the Golgi resident ATPase, Cod1p (Spf1p), contribute to Ca^2+^ homeostasis by sequestering Ca^2+^ into the lumen of ER/Golgi, an activity required for proper protein folding and transport through the secretory pathway [38–40]. We found that the deletion of the corresponding genes caused a reduction in cytosolic Hsp104p-GFP-associated aggregation (Fig. 2a). Cytosolic Ca^2+^ is regulated also by vacuolar pumps, including the Ca^2+^/H^+^ antiporter Vcx1p. Vcx1p is a low affinity/high capacity vacuolar antiporter in yeast cells [41]. Compared to wild-type cells, cells lacking *VCX1* display a significantly lower release of Ca^2+^ from intracellular stores when permeabilized in a medium lacking Ca^2+^ [42]. The Ca^2+^ ATPase Pmc1p, and the channel protein Yvc1p, release excess Ca^2+^ from the vacuole [41, 43]. Among these three proteins, the absence of vacuolar pumps Pmc1p* a*nd Yvc1p had no noticeable effect on cytosolic protein aggregation (Fig. 2a). In contrast, the absence of Vcx1p reduced aggregation to levels lower than that observed in wild-type cells (Fig. 2a). We noticed that the cells lacking both *vcx1*∆ and *pmc1*∆ resulted in more cells with cytosolic protein aggregates, likely due to excess Ca^2+^ in the cytosol. A similar increase in aggregation was observed when WT cells were treated with high levels of external Ca^2+^ (50 mM CaCl_2_ (Fig. 2a)).

Since the deletion *vcx1∆* resulted in reduced aggregation, we investigated if this vacuolar Ca^2+^/H^+^ antiporter affected CN signaling by measuring relative 4XCDRE-GFP levels corresponding to CN-activation. Interestingly, genetic ablation of *VCX1* resulted in marked activation of the CN-phosphatase (Fig. 2b). We also investigated the role of *VCX1* during ageing and observed that the cells lacking *VCX1* accumulated significantly fewer aggregates than the WT control irrespective of their age. Therefore, the data suggest that calcineurin activation achieved by depriving cells of *VCX1* boosts proteostasis during ageing (Fig. 2c).

### Calcineurin affects aggregate formation and aggregate clearance partly through the downstream target Crz1p

CN affects phosphorylation and activity of many downstream targets. Therefore, we tested whether the absence of any of these targets created a phenocopy of the absence of CN signaling (*cnb1∆*) to elucidate which CN-dependent pathway(s) are important for the proper management of protein aggregates. The CN-substrate proteins tested included Hph1p, Hph2p, Slm1p, Slm2p, Aly1p, Aly2p, and Crz1p. Among these, Aly1p and Slm1p/2p are involved in maintaining cytoskeleton integrity, membrane structure/function, and proper protein trafficking [44–48], whereas Hph1p/2p (Frt1p/2p) are ER-resident proteins involved in various stress responses, V-ATPase assembly, vacuole acidification, and Sec62p/63p-mediated translocation of specific proteins [49]. Lastly, *CRZ1* is a Ca^2+^/CN-requiring transcription factor that, when dephosphorylated by CN, activates an array of genes by binding to a conserved CN-dependent response element (CDREs) on various stress response genes [50–52].

**Fig. 3:**
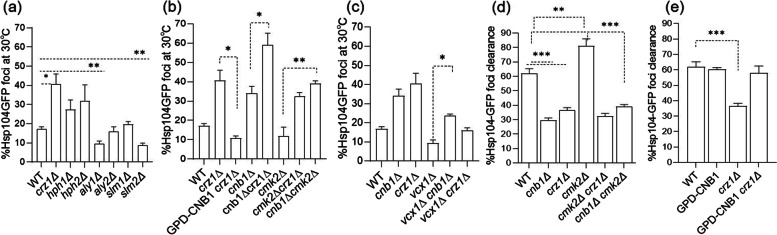
Calcineurin affects aggregate formation and aggregate clearance partly through the downstream target *CRZ1*. **a** Quantification of total cells containing Hsp104p-GFP aggregates in WT cells and in mutants lacking known direct substrates of the CN phosphatase as indicated. **b** Quantification of total cells containing Hsp104p-GFP associated aggregates in WT cells and in mutants with altered CN activity having *cnb1*Δ or *crz1*Δ as indicated. **c** Quantification of the total cells containing Hsp104p-GFP associated aggregates in WT cells and in the *vcx1*Δ cells when having altered CN activity having *cnb1*Δ or *crz1*Δ as indicated. **d** and **e** Quantification of the total cells containing Hsp104p-GFP associated aggregates in WT cells and in various mutants after letting them recover at 30 °C for 60 min following a heat shock at 38℃ (60 min). Data shown are the average of 3 independent ± SD. 200 cells from each independent experiment were counted and error bars indicate SD. *p < 0.05, **p < 0.005,***p < 0.0005, unpaired two-tailed t test

When testing mutants lacking any of these CN-dependent genes for aggregate formation in non–stress, Ca^2+^ replete, conditions we found that mutants lacking *hph1∆*, *hph2∆* and *crz1∆* displayed elevated formation of aggregates, of which cells lacking *crz1∆* had the strongest increase (Fig. 3a). In contrast, aggregation in *slm1∆* and *aly2∆* cells was indistinguishable from that of wild-type cells, whereas *slm2*∆ or *aly1*∆ cells displayed a somewhat reduced aggregation (Fig. 3a). Increased aggregation in cells lacking *hph1∆* or *hph2∆* may be the result of failures in vacuolar pH control as such failures have been shown previously to boost protein aggregation in the cytosol [53].

To test if the reduction in protein aggregation achieved by Cnb1p overproduction and a *CMK2* deletion (Fig. 3b) acted through the downstream transcription factor *CRZ1*, we overproduced Cnb1p and deleted *CMK2* in *crz1∆* cells. Interestingly, we found that the *CRZ1* deletion abolished the effect of the *CMK2* deletion whereas Cnb1p overproduction could still reduce aggregation in *crz1∆* cells (Fig. 3b and e). These results indicate that Cnb1p overproduction can affect PQC in a manner that is partly independent of *CRZ1*. In support of this notion, deleting *CRZ1* in *cnb1∆* cells resulted in an additive increase in protein aggregation (Fig. 3b). We were intrigued by the reduction of protein aggregation in cells lacking *VCX1* (Fig. 2a), and we therefore also determined if this reduction was dependent on CN signaling by removing *CNB1* or *CRZ1* in *vcx1∆* cells. Indeed, the reduced Hsp104p-GFP associated aggregation seen in *vcx1∆* cells was partly counteracted by removing *CNB1* or *CRZ1* (Fig. 3c), suggesting that the effect of Vcx1p deficiency on proteostasis is, at least partly, dependent on CN signaling.

We next tested whether the alterations observed in aggregate formation correlated with an altered capacity of disaggregation in cells with altered CN activity and, if so, this was dependent on *CRZ1*. For this, exponentially growing, unperturbed cells were shifted to 38℃ for 60 min and next allowed to recover at 30℃ for 60 min. In all mutant and wild-type strains analyzed, 100% of cells contained Hsp104p-associated aggregates after 60 min at 38 °C and disaggregation was scored by assessing the fraction of cells having cleared themselves of aggregates following 60 min recovery at 30 °C. First, we found that activating CN signaling by deleting *CMK2* accelerated aggregate clearance while Cnb1p-overproducing cells behaved like wild-type cells (Fig. 3d and e). Consistent with a role for CN in aggregate clearance, *cnb1∆* cells displayed a markedly retarded disaggregation. Similar to the reduced aggregate formation in the absence of stress, accelerated disaggregation achieved by deleting *CMK2* required the presence of *CRZ1* (Fig. 3d). In further agreement with the involvement of Crz1p in disaggregation, *crz1∆* cells, like *cnb1∆* cells, displayed a markedly reduced ability to clear themselves of aggregates (Fig. 3d). Deleting *CNB1* in *cmk2∆* cells strongly suppressed the accelerated clearance of aggregates, suggesting, again, that the beneficial effects on aggregate management achieved by deleting *CMK2* act through CN signaling. In contrast, the compromised disaggregation observed in *crz1∆* cells was strongly suppressed by overproducing Cnb1p, supporting the idea that *CNB1* overexpression and *CMK2* deletion act on partially different pathways to reduce Hsp104p-associated protein aggregates and that these pathways to a different degree depend on *CRZ1* (Fig. 3e).

### Overproducing calcineurin B represses α-Syn A53T toxicity

As shown herein, CN signaling greatly impacts on the cell’s ability to deal with protein aggregates. To elucidate a potential relevance, if any, of these findings in disease models in yeast, we analyzed the toxicity of the human disease protein, alpha-synuclein (α-Syn) of the Parkinson’s disease, in yeast cells with altered CN signaling. A significant hallmark of this disease is the aggregation of α-Syn, which is normally localized to the plasma membrane where it acts as an inhibitor of phospholipase D [54, 55]. Previous studies have demonstrated that the α-Syn protein causes cytotoxicity by interfering with metal ion transport, cell membrane properties, protein quality control, autophagy, and trafficking [21, 56–63].

**Fig. 4:**
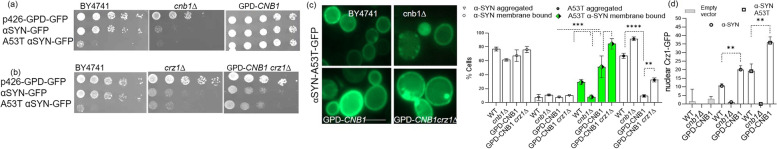
CN stimulation by calcineurin B overproduction represses α-Syn-A53T toxicity. **a** and **b** Representative drop test of from the indicated strains carrying α-Syn or α-Syn-A53T on a high copy number plasmid. A five-fold dilution for the control and indicated strains were spotted onto plates containing SD − Ura, and imaged after 72 h. **c** Yeast cells expressing GFP-fusions of α-Syn or α-Syn-A53T were visualized by fluorescence microscopy (right panel). α-Syn-A53T-GFP was localized in a puncta at the cell membrane and some scattered aggregates in the cytoplasm in wild-type cells (strain BY4741). In the cells overexpressing CNB1, α-Syn-A53T-GFP was evenly localized at the cell membrane only. In the crz1∆ cells overexpressing *CNB1*, α-Syn-A53T-GFP displayed an even distribution at the cell membrane and also formed large scattered inclusions on the cytoplasmic face of the cell membrane. Fraction of the cells plotted for the indicated strains expressing α-Syn or α-Syn-A53T-GFP (green) aggregates localizing to the cell membrane or dislocating to cytosolic puncta. **d** Percent fraction of the mutant strains indicated displaying nuclear localization of the transcription factor Crz1p-GFP in the strains expressing empty vector, α-Syn or α-Syn-A53T. Localization data shown in (**c** and **d**) are an average of 5 independent experiments ± SD. 200 cells from each independent experiment was counted and SD is depicted by the error bars. The scale bar represents 2 μm. *p < 0.05, **p < 0.005,***p < 0.0005, unpaired two-tailed t test

We tested the toxicity of wild-type α-Syn and α-SynA53T carrying a missense mutation associated with the familial form of Parkinson's generating postsynaptic deficits [64], using high copy number plasmids expressing GFP tagged and untagged α-Syn and α-Syn-A53T [54, 64–74]. The plasmids were propagated in the WT, *cnb1∆, GPD-CNB1*, and *crz1∆ GPD-CNB1* strains. A drop test growth analysis revealed that α-Syn is not particularly toxic in a WT yeast strain, whereas the mutant α-Syn-A53T is (Fig. 4a). In contrast, cells lacking *cnb1∆* were extremely sensitive to both α-Syn and α-Syn-A53T (Fig. 4a). Conversely, cells overexpressing *CNB1* strongly suppressed the toxic effect of α-Syn-A53T (Fig. 4a). Additionally, we tested if suppression of α-Syn-A53T toxicity mediated by Cnb1p overproduction required *CRZ1*. As shown, resistance towards α-Syn-A53T achieved by overproducing Cnb1p was partly dependent on the presence of *CRZ1* (compare Fig. 4a, right panel with Fig. 4b, right panel).

Previously, several studies utilizing yeast models of proteinopathy have shown that α-Syn predominantly localizes to the plasma membrane [54, 55, 75]. Primarily, α-Syn could be found in two states: a soluble unfolded monomeric form and a membrane-associated multimeric form, whereas the pathological state of the protein is either an oligomeric form and/or amyloid fibrils [76]. We found that WT cells expressing α-Syn-A53T-GFP displayed aggregated membrane-associated puncta along with some cytoplasmic aggregates whereas this localization was somewhat disrupted in the cells harboring *cnb1∆* (Fig. 4c). Interestingly, cells overexpressing *CNB1* displayed an enhanced localization of α-Syn-A53T-GFP to the cytosolic membrane and a concomitant decrease in its aggregation (Fig. 4c); phenotypes that were dependent on *CRZ1* (Fig. 4c). Following up on these results, we found that Cnb1p overproduction in cells carrying either of the α-Syn proteins boosted the nuclear localization of Crz1p-GFP [77] whereas this pattern was disrupted in cells lacking *CNB1* (Fig. 4d). Thus, the wild-type and A53T α-Syn alleles activate CN signaling to different extents in a manner influencing the degree of toxicity. Notably, the activation of CN signaling and Crz1p localization to the nucleus in the presence of α-Syn proteins was, in wild type cells, only able to overcome the toxicity of the less toxic α-Syn allele, whereas ectopic overproduction of Cnb1p overcame the toxicity of both alleles.

## Discussion

**Fig. 5:**
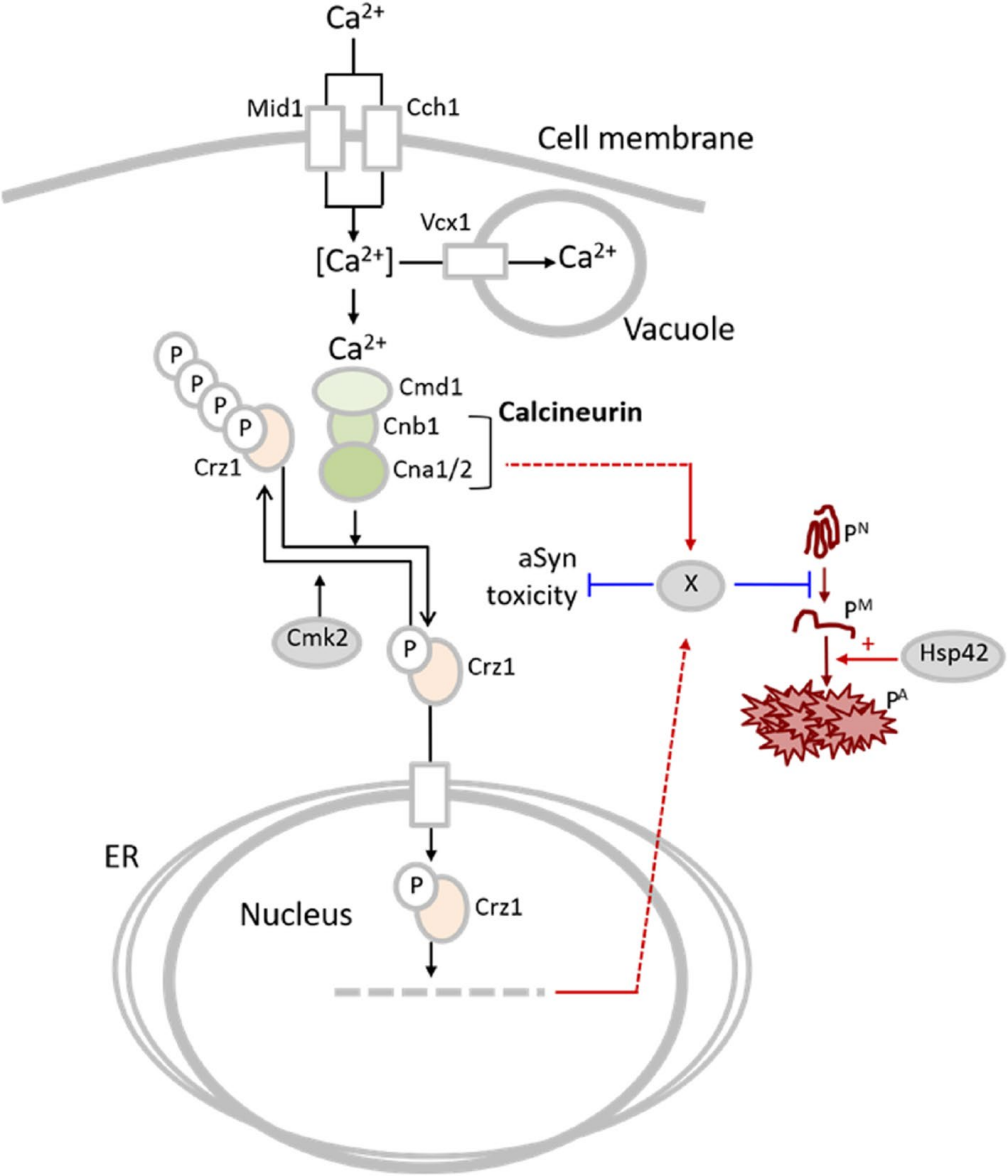
An overview of Calcineurin dependent bottlenecks regulating proteostasis in unstressed yeast. Activated calcineurin dephosphorylates the downstream transcription factor Crz1p stimulating the expression of target genes related to calcium homeostasis. Among these are channels that transport calcium into the ER, the vacuole or outside to restore homeostasis. Under calcium-replete conditions, normal calcineurin activity and balanced proteostasis are maintained by plasma membrane (Cch1p and Mid1p), vacuolar (Vcx1p and Pmc1p) and ER pumps (Pmr1p). The calmodulin-dependent kinase *CMK2* inhibits Crz1p activity by phosphorylating multiple amino acid residues. Upon stimulation by calcium, mutational perturbation of calcium signaling or aberrant proteostasis, calcium accumulates in the cytosol along with Hsp42p-dependent protein aggregates (PA). Calcineurin phosphatase stimulation, via an unknown effector (X), α-synuclein (α-Syn) toxicity and protein misfolding (pM) for native protein (pN)the latter of which suppresses protein aggregation. Where, enforced calcineurin activation by Cnb1p overproduction boosts proteostasis and suppresses α-Syn toxicity, suggesting that calcineurin activity is limiting for proteostasis under normal conditions. The T-shaped bars represent inhibition

In this study, we found that the CN phosphatase and its regulator, calcineurin B, prevents Hsp42p-dependent, Hsp104p-associated, aggregate formation in non-stressed, Ca^2+^-replete cells (Fig. 1). The fact that the aggregates formed in cells with compromised CN signalling required *HSP42* for their formation indicates that they are sorted spatially to the IPOD and Q-body (or Stress Foci) deposits/quality control compartments [24, 30–32]. We show also that activation of CN signalling, either by overproducing Cnb1p or deleting *CMK2*, a negative regulator of CN activity [33], causes a dramatic reduction in the cells containing Hsp104p-associated and guanylate kinase (guk1-7p)-associated aggregates, suggesting that CN signalling is limiting for protein quality control in non-stressed yeast cells. Similarly, suppression of aggregate formation and activation of CN signalling could be achieved by deleting the vacuolar Ca^2+^ influx pump *VCX1* suggesting that vacuolar Ca^2+^ storage is limiting CN activity, which, in turn, causes protein aggregation in the cytosol of non-stressed cells (Fig. 2). We, therefore, conclude that the balance between vacuolar Ca^2+^ storage capacity vs. free cytosolic [Ca^2+^] plays a pivotal role in determining the overall efficiency of the PQC machinery in non-stressed cells under calcium-replete conditions through the CN-dependent regulatory pathway (Fig. 5).

Deleting a well-studied CN client transcription factor, *CRZ1*, resulted in increased protein aggregation (Fig. 3a), which could not be repressed by deleting *CMK2*. Thus, the reduction in protein aggregation achieved by deleting *CMK2* in wild-type cells appears to act through *CRZ1*. In addition, accelerated clearance of protein aggregates achieved by deleting *CMK2* similarly required the *CRZ1* regulator (Fig. 3d). However, activation of CN signalling through overproduction of Cnb1p required *CRZ1* neither to reduce protein aggregation nor to accelerate aggregate clearance, suggesting that *CNB1* overexpression could function independently of *CRZ1* (Fig. 3b and e). Thus, it appears that signalling through *CNB1* functions in both *CRZ1-*dependent and -independent manners in cellular PQC.

The reduced aggregate formation observed in cells lacking the vacuolar influx pump *VCX1* was suppressed when *CNB1* was deleted (Fig. 3c), demonstrating that vacuolar Ca^2+^ influx is affecting PQC, at least partly, through CN signaling. It is possible also that the beneficial effects on PQC achieved by deleting *VCX1* acts through a post-transcriptional upregulation of another vacuolar Ca^2+^ channel, *PMC1* [33, 50, 78, 79]. Indeed, the reduced aggregation in *vcx1∆* cells were completely abolished when *PMC1* was concurrently deleted (Fig. 2a)*.* Such an enhanced aggregation could be due to Ca^2+^ overload in the cytoplasm resulting from the lack of vacuolar Ca^2+^ sequestration when both these vacuolar channels are absent, similar to a situation when cells were challenged with a high concentration of extracellular Ca^2+^ (Fig. 2a) (see also [42]).

*S. cerevisiae* has been adopted as a model for studying cellular and molecular aspects of Parkinson’s disease-associated α-Syn misfolding, aggregation, and toxicity. A distinct hallmark of this protein is that it forms inclusions bearing α-Syn protein monomers [54, 55] and several studies have shown that yeast cells overexpressing α-Syn display higher cytosolic Ca^2+^ levels [18, 21, 80, 81]. Therefore, we tested WT α-Syn and the missense mutation A53T associated with the familial form of Parkinson’s disease expressed from a high copy number plasmid [54, 82] in various yeast mutants known to affect calcium homeostasis and signalling. Interestingly, only the cells overexpressing *CNB1* strongly suppressed the toxic effects of both α-Syn and its variant A53T. Conversely, yeast cells harbouring *cnb1∆* displayed enhanced sensitivity to both α-Syn and its variant A53T. Suppression of α-Syn and A53T α-Syn toxicity achieved by overexpressing *CNB1* correlated with a normal membrane localization of the disease protein and, simultaneously, a reduced cytosolic aggregation (Fig. 4c). This indicates that in a yeast model of Parkinson’s disease and overall proteostasis, enhanced CN signalling by Cnb1p overproduction affects properties of α-Syn that have previously been linked to the disease-associated pathological effects (Fig. 5). Our findings suggest that elucidating to which extent aberrant CN signaling is contributing to the progression of Parkinson’s disease, and perhaps other proteinopathies, may be warranted.

## Materials and methods

### Yeast strain construction

The various knockout mutants used were constructed in BY4741 by integrating the gene::HphNT1 hygromycin cassette amplified using the pYM20 integrative module [83] enlisted in Table 1. The GPD promoter was amplified from the pYM-N15 plasmid [83] and incorporated upstream of the *CNB1* ORF in the WT BY4741 and in CNB1-GFP. The C-terminal EGFP-HIS3-MX cassette was amplified from the pYM-28 plasmid [83] and was incorporated in frame with the *ORF of CRZ1 and the HSP104 respectively*. For removing GFP from α-Syn-GFP and A53T-GFP variants *ClaI-XhoI* restriction digestion was performed and the linearized pRS426 GPD harboring the α-Syn and A53T variants of α-Syn on the plasmids were re-ligated.

**Table 1:** *S. cerevisiae* strains used in this study

Name		Description	Source
BY4741		*MATa his3Δ1 leu2Δ0 met15Δ0 ura3Δ0*	Invitrogen Inc
		BY4741 *cna1Δ::kanMX4*	Invitrogen Inc
		BY4741 *cna2Δ::kanMX4*	Invitrogen Inc
		BY4741 *cnb1Δ::kanMX4*	Invitrogen Inc
		BY4741 *cmk2Δ::kanMX4*	Invitrogen Inc
		BY4741 *hsp42Δ::kanMX4*	Invitrogen Inc
		BY4741 *cmd1-3::kanMX4*	Invitrogen Inc
		BY4741 *cod1Δ::kanMX4*	Invitrogen Inc
		BY4741 *cch1Δ::kanMX4*	Invitrogen Inc
		BY4741 *mid1Δ::kanMX4*	Invitrogen Inc
		BY4741 *cmk2Δ::kanMX4*	Invitrogen Inc
		BY4741 *crz1Δ::kanMX4*	Invitrogen Inc
		BY4741 *vcx1Δ::kanMX4*	Invitrogen Inc
		BY4741 *pmc1Δ::kanMX4*	Invitrogen Inc
		BY4741 *pmr1Δ::kanMX4*	Invitrogen Inc
		BY4741 *yvc1Δ::kanMX4*	Invitrogen Inc
		BY4741 *hph1Δ::kanMX4*	Invitrogen Inc
		BY4741 *hph2Δ::kanMX4*	Invitrogen Inc
		BY4741 *aly1Δ::kanMX4*	Invitrogen Inc
		BY4741 *aly2Δ::kanMX4*	Invitrogen Inc
		BY4741 *slm1Δ::kanMX4*	Invitrogen Inc
		BY4741 *slm2Δ::kanMX4*	Invitrogen Inc
		BY4741 *CNB1-GFP::HIS3-MX6*	EUROSCARF
		BY4741 *natMX6::pGPD-CNB1*	This study
		BY4741 *natMX6::pGPD-CNB1 GFP-HIS3-MX6*	This study
		BY4741 *natMX6::pGPD-CNB1 crz1Δ::hph1NT1*	This study
		BY4741 *vcx1Δ::hphNT1 pmc1Δ::kanMX4*	This study
		BY4741 *CRZ1-eGFP-HIS3MX6*	This study
		BY4741 *CRZ1-eGFP-HIS3MX6 cnb1Δ::hph1NT1*	This study
		BY4741 *CRZ1-eGFP-HIS3MX6 natMX6::pGPD-CNB1*	This study
	WT-H	MATa *his3Δ1 leu2Δ0 met15Δ0 ura3Δ0 HSP104-GFP-HIS3MX6*	This study
		WT-H *cnb1Δ::kanMX4*	This study
		WT-H *crz1Δ::kanMX4*	This study
		WT-H *cna1Δ::kanMX4 cna2Δ::hph1NT1*	This study
		WT-H *cnb1Δ::kanMX4 cmk2Δ::hph1NT1*	This study
		WT-H *crz1Δ::kanMX4 cmk2Δ::hph1NT1*	This study
		WT-H guk1-7-mscarlet	This study
		WT-H *natMX6:pGPD-CNB1*	This study
		WT-H *cch1Δ::kanMX4 mid1Δ::hph1NT1*	This study
		WT-H *yvc1Δ::hph1NT1*	This study
		WT-H *vcx1Δ::hph1NT1*	This study
		WT-H *pmc1Δ::hph1NT1*	This study
		WT-H *pmr1Δ::hph1NT1*	This study
		WT-H *vcx1Δ::hph1NT1 cnb1Δ::kanMX4*	This study
		WT-H *vcx1Δ::hph1NT1 crz1Δ::kanMX4*	This study
		WT-H *cnb1Δ::kanMX4 crz1Δ::hph1NT1*	This study
		WT-H *vcx1Δ::hph1NT1 pmc1Δ::kanMX4*	This study
		WT-H *natMX6:pGPD-CNB1 crz1Δ::hph1NT1*	This study
		WT-H *hph1Δ::kanMX4*	This study
		WT-H *hph2Δ::kanMX4*	This study
		WT-H *aly1Δ::kanMX4*	This study
		WT-H *aly2Δ::kanMX4*	This study
		WT-H *slm1Δ::kanMX4*	This study
		WT-H *slm2Δ::kanMX4*	This study

### Protein aggregation visualization and aggregate clearance analysis

As previously described [24], cells were grown in medium supplemented with 2% glucose at permissive temperature (30 °C/22 °C) until the mid-exponential phase (OD_600_ 0.5). Where indicated, Cyclosporin A from (Sigma) and FK506 (LC Laboratories, Woburn MA) were used at 1 μg/mL and CaCl_2_ (Sigma-Aldrich, St. Louis, MO) was used at 50 mM). Protein aggregation was also induced by shifting the cells to 38 °C for 60 min or by allowing cells to age replicatively at 30 °C. Cells were fixed in 3.7% final concentration formaldehyde and washed with PBS. Z-stack images were acquired using a conventional fluorescence microscope; a Zeiss Axio Observer Z1 inverted microscope equipped with an Axiocam 506 camera and a Plan-Apochromat 100x/1.40 NA Oil DIC M27 objective. Images were quantified by manually counting the fraction of cells containing aggregates in the ImageJ software. All the data are based on the average of at least three individual experiments, with error bars representing SD.

### Statistical analysis

Data from quantifications are presented as the average of ≥ 3 biological experiments ± SEM unless indicated otherwise. Aggregate quantifications of cells are based on counting 200 or more cells per strain at each condition for three biological replicates. Data analysis and visualization were performed with GraphPad Prism 8.3.1. In figures, asterisks denote statistical significance determined by the indicated statistical test with ns *p* > 0.05, *p ≤ 0.05, **p ≤ 0.01, ***p ≤ 0.001, ****p < 0.0001.

### Co-localization analysis

Cells with the indicated fluorescent protein reporters were grown in either YPD or SCD as indicated supplemented with 2% glucose at a permissive temperature (30˚C) until the mid-exponential phase (OD_600_ 0.5).

### Gel electrophoresis and immunoblotting

Cells were harvested in exponential phase at an OD_600_ of 0.8, as previously described [29]. A total of 6.0 OD_600_ cells were then lysed in 0.2 M NaOH, supplemented by complete™ Protease Inhibitor Cocktail (Roche) and Halt™ Phosphatase Inhibitor Cocktail (Fischer scientific), incubated for 20 min on ice, pelleted, resuspended in 50–70 μl sample buffer (1 × Laemmli buffer (80 mM Tris–HCL pH 6.8, 10 mM EDTA pH 8.0, 8% SDS, 20% Glycerol, 0.004% Bromophenol blue), 8 M Urea, 2.5% β-Mercaptoethanol) and heated for 5.0 min at 90 °C. About 15 μl of the protein sample was loaded per lane of a 12% Criterion TGX Precast Midi Protein Gel (Bio-Rad, Hercules, CA, USA), blotted onto Trans-Blot Turbo Midi Nitrocellulose Transfer Packs (Bio-Rad, Hercules, CA, USA), and probed overnight with primary antibodies: Membranes were probed with anti-GFP (ab6556; Abcam). As secondary antibodies, goat anti-mouse IRDye 800CW and goat anti-rabbit IRDye 680 (LI-COR; 1/20000 dilution) were used. Membranes were scanned using the LI-COR Odyssey Infrared scanner. Images were then quantified by the ImageJ software.

### Drop tests on agar plates

Strains with the indicated plasmids were grown in the indicated media (YPD or SCD) supplemented with amino acids along with 2% glucose at the permissive temperature (30 °C/22 °C) until the mid-exponential phase (OD_600_ 0.7). The culture ODs were adjusted to 0.5 on YPD and SCD (OD_600_ ~ 1.0) for serial dilutions and then spotted on agar plates for colony formation. The cell suspensions were spotted onto the respective drop-out media plates and incubated at the indicated temperatures for 2–3 days.

### Old cell isolation

Old cells were obtained using the magnetic beads biotin-streptavidin system according to established protocols [35]. Cells were grown to exponential phase at 30 °C, approximately 3 × 10^7^ cells were harvested, washed in PBS and then labelled with EZ link Sulfo-NHS-LC biotin (Thermo Scientific) at a final concentration of 0.5 mg/mL in 1 ml. Excess biotin was washed away, and cells were resuspended in 1L growth medium and cultured for about 15 h overnight at 30 °C, using an orbital shaker (180 rpm). When the cultures had reached an OD_600_ of about 0.5, cells were washed with PBS and incubated with 0.05 mg/ml MagnaBind streptavidin beads (Thermo Scientific). Biotin-labelled cells were then isolated using a magnetic sorter and continuous washes with PBS containing 0.5% glucose. Cells were resuspended in a 7 ml growth medium and used directly for subsequent experiments. The median age of the old cells was determined by counting bud scars in z-stack images upon staining cells with 10 µg/mL Wheat Germ Agglutinin (WGA, Thermo Scientific).

### Determination of calcineurin activity

Calcineurin activity was determined using a reporter plasmid pAMS366- 4xCDRE-GFP described in [29]. Briefly, at about an OD_600_ of 2.0, yeast cells were harvested and then lysed in 0.2 M NaOH supplemented by complete™ Protease Inhibitor Cocktail (Roche) and Halt™ Phosphatase Inhibitor Cocktail (Fischer scientific), incubated for 20 min on ice, pelleted, resuspended in 50–70 μl sample buffer (1 × Laemmli buffer (80 mM Tris–HCL pH 6.8, 10 mM EDTA pH 8.0, 8% SDS, 20% Glycerol, 0.004% Bromophenol blue), 8 M Urea, 2.5% β-Mercaptoethanol), and heated for 5.0 min at 90 °C. The lysates were snap-chilled and spun for one minute and about 15 μl of the sample was loaded on the SDS-PAGE.

## Data Availability

All data are contained within this article.

## References

[1] Viladevall L, Serrano R, Ruiz A, Domenech G, Giraldo J, Barcelo A (2004). Characterization of the calcium-mediated response to alkaline stress in Saccharomyces cerevisiae. J Biol Chem.

[2] Cunningham KW (2011). Acidic calcium stores of Saccharomyces cerevisiae. Cell Calcium.

[3] Cyert MS, Philpott CC (2013). Regulation of cation balance in Saccharomyces cerevisiae. Genetics.

[4] Dolmetsch RE, Lewis RS, Goodnow CC, Healy JI (1997). Differential activation of transcription factors induced by Ca2+ response amplitude and duration. Nature.

[5] Berridge MJ, Bootman MD, Roderick HL (2003). Calcium signalling: dynamics, homeostasis and remodelling. Nat Rev Mol Cell Biol.

[6] Goldman A, Roy J, Bodenmiller B, Wanka S, Landry CR, Aebersold R (2014). The calcineurin signaling network evolves via conserved kinase-phosphatase modules that transcend substrate identity. Mol Cell.

[7] Arsenault HE, Roy J, Mapa CE, Cyert MS, Benanti JA (2015). Hcm1 integrates signals from Cdk1 and calcineurin to control cell proliferation. Mol Biol Cell.

[8] Guiney EL, Goldman AR, Elias JE, Cyert MS (2015). Calcineurin regulates the yeast synaptojanin Inp53/Sjl3 during membrane stress. Mol Biol Cell.

[9] Saneyoshi T, Kume S, Amasaki Y, Mikoshiba K (2002). The Wnt/calcium pathway activates NF-AT and promotes ventral cell fate in Xenopus embryos. Nature.

[10] Nishiyama T, Yoshizaki N, Kishimoto T, Ohsumi K (2007). Transient activation of calcineurin is essential to initiate embryonic development in Xenopus laevis. Nature.

[11] Dwivedi M, Song HO, Ahnn J (2009). Autophagy genes mediate the effect of calcineurin on life span in C. elegans. Autophagy.

[12] Mair W, Morantte I, Rodrigues AP, Manning G, Montminy M, Shaw RJ (2011). Lifespan extension induced by AMPK and calcineurin is mediated by CRTC-1 and CREB. Nature.

[13] Nakai Y, Horiuchi J, Tsuda M, Takeo S, Akahori S, Matsuo T (2011). Calcineurin and its regulator sra/DSCR1 are essential for sleep in Drosophila. J Neurosci.

[14] Lee JI, Mukherjee S, Yoon KH, Dwivedi M, Bandyopadhyay J (2013). The multiple faces of calcineurin signaling in Caenorhabditis elegans: development, behaviour and aging. J Biosci.

[15] Kujawski S, Lin W, Kitte F, Bormel M, Fuchs S, Arulmozhivarman G (2014). Calcineurin regulates coordinated outgrowth of zebrafish regenerating fins. Dev Cell.

[16] Deng H, Gerencser AA, Jasper H (2015). Signal integration by Ca(2+) regulates intestinal stem-cell activity. Nature.

[17] Caraveo G, Auluck PK, Whitesell L, Chung CY, Baru V, Mosharov EV (2014). Calcineurin determines toxic versus beneficial responses to alpha-synuclein. Proc Natl Acad Sci U S A.

[18] Angelova PR, Ludtmann MH, Horrocks MH, Negoda A, Cremades N, Klenerman D (2016). Ca2+ is a key factor in alpha-synuclein-induced neurotoxicity. J Cell Sci.

[19] Lieberman OJ, Choi SJ, Kanter E, Saverchenko A, Frier MD, Fiore GM, et al. α-Synuclein-Dependent Calcium Entry Underlies Differential Sensitivity of Cultured SN and VTA Dopaminergic Neurons to a Parkinsonian Neurotoxin. eNeuro. 2017;4(6):ENEURO.0167-17.2017. 10.1523/ENEURO.0167-17.2017 PMC5701296 29177188

[20] Betzer C, Jensen PH (2018). Reduced cytosolic calcium as an early decisive cellular state in Parkinson's disease and synucleinopathies. Front Neurosci.

[21] Habernig L, Broeskamp F, Aufschnaiter A, Diessl J, Peselj C, Urbauer E (2021). Ca2+ administration prevents alpha-synuclein proteotoxicity by stimulating calcineurin-dependent lysosomal proteolysis. PLoS Genet.

[22] Erjavec N, Larsson L, Grantham J, Nystrom T (2007). Accelerated aging and failure to segregate damaged proteins in Sir2 mutants can be suppressed by overproducing the protein aggregation-remodeling factor Hsp104p. Genes Dev.

[23] Spokoini R, Moldavski O, Nahmias Y, England JL, Schuldiner M, Kaganovich D (2012). Confinement to organelle-associated inclusion structures mediates asymmetric inheritance of aggregated protein in budding yeast. Cell Rep.

[24] Babazadeh R, Ahmadpour D, Jia S, Hao X, Widlund P, Schneider K (2019). Syntaxin 5 is required for the formation and clearance of protein inclusions during proteostatic stress. Cell Rep.

[25] Schneider KL, Ahmadpour D, Keuenhof KS, Eisele-Burger AM, Berglund LL, Eisele F (2022). Using reporters of different misfolded proteins reveals differential strategies in processing protein aggregates. J Biol Chem.

[26] Comyn SA, Young BP, Loewen CJ, Mayor T (2016). Prefoldin promotes proteasomal degradation of cytosolic proteins with missense mutations by maintaining substrate solubility. PLoS Genet.

[27] Kingsbury TJ, Cunningham KW (2000). A conserved family of calcineurin regulators. Genes Dev.

[28] Geiser JR, van Tuinen D, Brockerhoff SE, Neff MM, Davis TN (1991). Can calmodulin function without binding calcium?. Cell.

[29] Diessl J, Nandy A, Schug C, Habernig L, Büttner S. Stable and destabilized GFP reporters to monitor calcineurin activity in Saccharomyces cerevisiae. Microb Cell. 2020;7(4):106–14. 10.15698/mic2020.04.713. 10.15698/mic2020.04.713 PMC7136757 32274389

[30] Kaganovich D, Kopito R, Frydman J (2008). Misfolded proteins partition between two distinct quality control compartments. Nature.

[31] Specht S, Miller SB, Mogk A, Bukau B (2011). Hsp42 is required for sequestration of protein aggregates into deposition sites in Saccharomyces cerevisiae. J Cell Biol.

[32] Malinovska L, Kroschwald S, Munder MC, Richter D, Alberti S (2012). Molecular chaperones and stress-inducible protein-sorting factors coordinate the spatiotemporal distribution of protein aggregates. Mol Biol Cell.

[33] Xu H, Fang T, Yan H, Jiang L (2019). The protein kinase Cmk2 negatively regulates the calcium/calcineurin signalling pathway and expression of calcium pump genes PMR1 and PMC1 in budding yeast. Cell Commun Signal.

[34] Smeal T, Claus J, Kennedy B, Cole F, Guarente L (1996). Loss of transcriptional silencing causes sterility in old mother cells of S. cerevisiae. Cell.

[35] Sinclair DA, Guarente L (2014). Small-molecule allosteric activators of sirtuins. Annu Rev Pharmacol Toxicol.

[36] Eilam Y, Lavi H, Grossowicz N (1985). Mechanism of stimulation of Ca2+ uptake by miconazole and ethidium bromide in yeasts: role of vacuoles in Ca2+ detoxification. Microbios.

[37] Dunn T, Gable K, Beeler T (1994). Regulation of cellular Ca2+ by yeast vacuoles. J Biol Chem.

[38] Sorin A, Rosas G, Rao R (1997). PMR1, a Ca2+-ATPase in yeast Golgi, has properties distinct from sarco/endoplasmic reticulum and plasma membrane calcium pumps. J Biol Chem.

[39] Durr G, Strayle J, Plemper R, Elbs S, Klee SK, Catty P (1998). The medial-Golgi ion pump Pmr1 supplies the yeast secretory pathway with Ca2+ and Mn2+ required for glycosylation, sorting, and endoplasmic reticulum-associated protein degradation. Mol Biol Cell.

[40] Strayle J, Pozzan T, Rudolph HK (1999). Steady-state free Ca(2+) in the yeast endoplasmic reticulum reaches only 10 microM and is mainly controlled by the secretory pathway pump pmr1. EMBO J.

[41] Cagnac O, Aranda-Sicilia MN, Leterrier M, Rodriguez-Rosales MP, Venema K (2010). Vacuolar cation/H+ antiporters of Saccharomyces cerevisiae. J Biol Chem.

[42] Callewaert G, D'Hooge P, Ma TY, Del Vecchio M, Van Eyck V, Franssens V (2020). Decreased vacuolar Ca(2+) storage and disrupted vesicle trafficking underlie alpha-synuclein-induced Ca(2+) dysregulation in S. cerevisiae. Front Genet.

[43] Denis V, Cyert MS (2002). Internal Ca(2+) release in yeast is triggered by hypertonic shock and mediated by a TRP channel homologue. J Cell Biol.

[44] O'Donnell AF, Apffel A, Gardner RG, Cyert MS (2010). Alpha-arrestins Aly1 and Aly2 regulate intracellular trafficking in response to nutrient signaling. Mol Biol Cell.

[45] O'Donnell AF, Huang L, Thorner J, Cyert MS (2013). A calcineurin-dependent switch controls the trafficking function of alpha-arrestin Aly1/Art6. J Biol Chem.

[46] Berchtold D, Piccolis M, Chiaruttini N, Riezman I, Riezman H, Roux A (2012). Plasma membrane stress induces relocalization of Slm proteins and activation of TORC2 to promote sphingolipid synthesis. Nat Cell Biol.

[47] Bultynck G, Heath VL, Majeed AP, Galan JM, Haguenauer-Tsapis R, Cyert MS (2006). Slm1 and slm2 are novel substrates of the calcineurin phosphatase required for heat stress-induced endocytosis of the yeast uracil permease. Mol Cell Biol.

[48] Heath VL, Shaw SL, Roy S, Cyert MS (2004). Hph1p and Hph2p, novel components of calcineurin-mediated stress responses in Saccharomyces cerevisiae. Eukaryot Cell.

[49] Pina FJ, O'Donnell AF, Pagant S, Piao HL, Miller JP, Fields S (2011). Hph1 and Hph2 are novel components of the Sec63/Sec62 posttranslational translocation complex that aid in vacuolar proton ATPase biogenesis. Eukaryot Cell.

[50] Matheos DP, Kingsbury TJ, Ahsan US, Cunningham KW (1997). Tcn1p/Crz1p, a calcineurin-dependent transcription factor that differentially regulates gene expression in Saccharomyces cerevisiae. Genes Dev.

[51] Stathopoulos AM, Cyert MS (1997). Calcineurin acts through the CRZ1/TCN1-encoded transcription factor to regulate gene expression in yeast. Genes Dev.

[52] Stathopoulos-Gerontides A, Guo JJ, Cyert MS (1999). Yeast calcineurin regulates nuclear localization of the Crz1p transcription factor through dephosphorylation. Genes Dev.

[53] Hill SM, Hao X, Gronvall J, Spikings-Nordby S, Widlund PO, Amen T (2016). Asymmetric inheritance of aggregated proteins and age reset in yeast are regulated by Vac17-dependent vacuolar functions. Cell Rep.

[54] Outeiro TF, Lindquist S (2003). Yeast cells provide insight into alpha-synuclein biology and pathobiology. Science.

[55] Dixon C, Mathias N, Zweig RM, Davis DA, Gross DS (2005). Alpha-synuclein targets the plasma membrane via the secretory pathway and induces toxicity in yeast. Genetics.

[56] Willingham S, Outeiro TF, DeVit MJ, Lindquist SL, Muchowski PJ (2003). Yeast genes that enhance the toxicity of a mutant huntingtin fragment or alpha-synuclein. Science.

[57] Bussell R, Eliezer D (2003). A structural and functional role for 11-mer repeats in alpha-synuclein and other exchangeable lipid binding proteins. J Mol Biol.

[58] Liang J, Clark-Dixon C, Wang S, Flower TR, Williams-Hart T, Zweig R (2008). Novel suppressors of alpha-synuclein toxicity identified using yeast. Hum Mol Genet.

[59] Zabrocki P, Bastiaens I, Delay C, Bammens T, Ghillebert R, Pellens K (2008). Phosphorylation, lipid raft interaction and traffic of alpha-synuclein in a yeast model for Parkinson. Biochim Biophys Acta.

[60] Yeger-Lotem E, Riva L, Su LJ, Gitler AD, Cashikar AG, King OD (2009). Bridging high-throughput genetic and transcriptional data reveals cellular responses to alpha-synuclein toxicity. Nat Genet.

[61] Ruiperez V, Darios F, Davletov B (2010). Alpha-synuclein, lipids and Parkinson's disease. Prog Lipid Res.

[62] Lashuel HA, Overk CR, Oueslati A, Masliah E (2013). The many faces of alpha-synuclein: from structure and toxicity to therapeutic target. Nat Rev Neurosci.

[63] Galvagnion C (2017). The role of lipids interacting with alpha-synuclein in the pathogenesis of Parkinson's disease. J Parkinsons Dis.

[64] Polymeropoulos MH, Lavedan C, Leroy E, Ide SE, Dehejia A, Dutra A (1997). Mutation in the alpha-synuclein gene identified in families with Parkinson's disease. Science.

[65] Kruger R, Kuhn W, Muller T, Woitalla D, Graeber M, Kosel S (1998). Ala30Pro mutation in the gene encoding alpha-synuclein in Parkinson's disease. Nat Genet.

[66] Singleton AB, Farrer M, Johnson J, Singleton A, Hague S, Kachergus J (2003). alpha-Synuclein locus triplication causes Parkinson's disease. Science.

[67] Chartier-Harlin MC, Kachergus J, Roumier C, Mouroux V, Douay X, Lincoln S (2004). Alpha-synuclein locus duplication as a cause of familial Parkinson's disease. Lancet.

[68] Zarranz JJ, Alegre J, Gomez-Esteban JC, Lezcano E, Ros R, Ampuero I (2004). The new mutation, E46K, of alpha-synuclein causes Parkinson and Lewy body dementia. Ann Neurol.

[69] Tofaris GK, Spillantini MG (2007). Physiological and pathological properties of alpha-synuclein. Cell Mol Life Sci.

[70] Auluck PK, Caraveo G, Lindquist S (2010). alpha-Synuclein: membrane interactions and toxicity in Parkinson's disease. Annu Rev Cell Dev Biol.

[71] Fauvet B, Fares MB, Samuel F, Dikiy I, Tandon A, Eliezer D (2012). Characterization of semisynthetic and naturally Nalpha-acetylated alpha-synuclein in vitro and in intact cells: implications for aggregation and cellular properties of alpha-synuclein. J Biol Chem.

[72] Appel-Cresswell S, Vilarino-Guell C, Encarnacion M, Sherman H, Yu I, Shah B (2013). Alpha-synuclein p.H50Q, a novel pathogenic mutation for Parkinson's disease. Mov Disord.

[73] Lesage S, Anheim M, Letournel F, Bousset L, Honore A, Rozas N (2013). G51D alpha-synuclein mutation causes a novel parkinsonian-pyramidal syndrome. Ann Neurol.

[74] Ghiglieri V, Calabrese V, Calabresi P (2018). Alpha-synuclein: from early synaptic dysfunction to neurodegeneration. Front Neurol.

[75] Soper JH, Roy S, Stieber A, Lee E, Wilson RB, Trojanowski JQ (2008). Alpha-synuclein-induced aggregation of cytoplasmic vesicles in Saccharomyces cerevisiae. Mol Biol Cell.

[76] Burre J, Sharma M, Sudhof TC (2015). Definition of a molecular pathway mediating alpha-synuclein neurotoxicity. J Neurosci.

[77] Boustany LM, Cyert MS (2002). Calcineurin-dependent regulation of Crz1p nuclear export requires Msn5p and a conserved calcineurin docking site. Genes Dev.

[78] Cunningham KW, Fink GR (1996). Calcineurin inhibits VCX1-dependent H+/Ca2+ exchange and induces Ca2+ ATPases in Saccharomyces cerevisiae. Mol Cell Biol.

[79] Cyert MS (2001). Genetic analysis of calmodulin and its targets in Saccharomyces cerevisiae. Annu Rev Genet.

[80] Quist A, Doudevski I, Lin H, Azimova R, Ng D, Frangione B (2005). Amyloid ion channels: a common structural link for protein-misfolding disease. Proc Natl Acad Sci U S A.

[81] Schmidt F, Levin J, Kamp F, Kretzschmar H, Giese A, Botzel K (2012). Single-channel electrophysiology reveals a distinct and uniform pore complex formed by alpha-synuclein oligomers in lipid membranes. PLoS ONE.

[82] Poewe W, Seppi K, Tanner CM, Halliday GM, Brundin P, Volkmann J (2017). Parkinson disease. Nat Rev Dis Primers.

[83] Janke C, Magiera MM, Rathfelder N, Taxis C, Reber S, Maekawa H (2004). A versatile toolbox for PCR-based tagging of yeast genes: new fluorescent proteins, more markers and promoter substitution cassettes. Yeast.

